# Depressive symptom patterns in older adults: evidence from two national ageing cohorts

**DOI:** 10.3389/fpsyt.2026.1893371

**Published:** 2026-07-10

**Authors:** Qihao Wang, Yikang Shen, Mingrui Liu, Yaning Li, Haiyu Liu, Yufeng Zhao

**Affiliations:** Data Center for Traditional Chinese Medicine, China Academy of Chinese Medical Sciences, Beijing, China

**Keywords:** ageing cohorts, CES-D, depressive symptoms, late-life depression, latent class analysis, risk stratification

## Abstract

**Background:**

Late-life depressive symptoms are heterogeneous, and total symptom scores may obscure symptom configurations with distinct prognostic implications. We aimed to identify reproducible depressive symptom patterns in older adults and test whether these patterns added information beyond CES-D total score.

**Methods:**

We conducted a harmonised longitudinal cohort analysis using the Health and Retirement Study (HRS) as the discovery cohort and the English Longitudinal Study of Ageing (ELSA) as an independent replication cohort. Baseline was each participant’s first wave with complete CES-D-8 item data and sufficient information to construct five harmonised symptom domains. Latent class analysis was used to derive symptom patterns. Cross-cohort structural replication, baseline external validity, longitudinal associations and incremental value beyond baseline CES-D total score were evaluated.

**Results:**

The main LCA sample included 43,091 HRS participants and 19,037 ELSA participants. A 4-class solution was selected in both cohorts: Minimal Symptoms, Somatic-Motivational, Affective-Social and Global Distress. The active symptom patterns showed cross-cohort structural correspondence, with item-probability correlations of 0.980 for Somatic-Motivational, 0.828 for Affective-Social and 0.860 for Global Distress. Baseline external-validity profiles were also similar across cohorts. After adjustment for baseline CES-D total score, the Somatic-Motivational pattern was the most consistent prognostic pattern, showing higher odds of future elevated depressive symptom status in ELSA (OR, 1.39; 95% CI, 1.22-1.58) and HRS (OR, 1.53; 95% CI, 1.41-1.65), and higher risk of ADL worsening and mortality in both cohorts. Adding symptom pattern to score-only models improved likelihood-based fit across outcomes and identified observed risks above score-based expectations in the Somatic-Motivational pattern.

**Conclusions:**

Reproducible depressive symptom patterns can be identified across two national ageing cohorts. The Somatic-Motivational pattern retained prognostic information beyond total depressive symptom severity, supporting symptom configuration as a complementary layer of late-life risk assessment.

## 1 Introduction

Depressive symptoms in later life are a major public health concern because clinically relevant depressive symptom burden is common in older populations, even when the prevalence of major depressive disorder is lower than in younger adults (1–3). Late-life depression is also clinically consequential, with evidence linking it to functional disability, reduced quality of life, cognitive impairment, dementia risk, suicide risk and mortality (2, 4–7). Its clinical presentation is heterogeneous: older adults with depression may show affective distress, anhedonia, somatic symptoms, sleep disturbance, cognitive changes, loneliness, fatigue or motivational difficulty in different combinations (2, 4, 5). Authoritative reviews of depression have highlighted that this heterogeneity is one reason precision medicine and more refined patient stratification are needed in depressive disorders (8). This heterogeneity also matters for population ageing research because symptom patterns that look similar on a total severity scale may differ in clinical context and subsequent health risk (8, 9).

Most large ageing cohorts measure depressive symptoms using brief self-report instruments and summarise responses as a total score (10). The Center for Epidemiologic Studies Depression scale (CES-D) was designed for epidemiologic research and remains widely used because it provides a practical measure of depressive symptom burden in population studies (11). However, the CES-D was not conceptually limited to a single homogeneous construct; its items cover negative affect, positive affect, somatic or motivational symptoms and interpersonal or social-affective content (11, 12). Reviews and factor-analytic work support the multidimensional nature of CES-D symptoms, which suggests that symptom configuration may contain information that is lost when all items are collapsed into one total score.

The limitation of relying only on symptom sums is not unique to the CES-D (9). Methodological and clinical work on depression has argued that total scores can obscure important item-level variation because different symptoms may have different correlates, causes, functional consequences and prognostic meanings (13). This concern is especially relevant in older adults, for whom fatigue, poor sleep, social isolation and functional limitation may reflect both depression-related processes and ageing-related health changes (14). Therefore, a symptom-pattern approach can complement severity scoring by asking whether groups of older adults show reproducible configurations of affective, motivational, sleep and social symptoms.

Latent class analysis provides a person-centred framework for identifying subgroups with similar patterns across categorical symptom indicators (15, 16). Prior studies have used latent or cluster-based approaches to describe heterogeneity in late-life depressive symptom profiles, supporting the clinical plausibility of symptom-pattern phenotyping (13). Nevertheless, a symptom class solution is not automatically useful simply because it is statistically identifiable (15, 17). To support clinical and epidemiologic interpretation, symptom patterns should be reproducible across independent cohorts, externally valid with respect to baseline health and functional profiles, and prospectively associated with later outcomes beyond established severity measures (17, 18).

This evidence gap is important because many previous symptom-profile analyses have been based on a single dataset, a clinically restricted or threshold-defined sample, or have not tested whether the resulting classes add information beyond a depressive symptom total score (18). In longitudinal ageing research, external validation across cohorts is particularly valuable because survey design, population composition and measurement context can influence both symptom endorsement and baseline health profiles (18–20). For prediction and risk-stratification purposes, it is also necessary to test whether a new phenotype improves model fit, calibration or risk separation after accounting for conventional predictors such as baseline depressive symptom severity. These considerations motivate a discovery-validation design rather than a single-cohort clustering exercise.

In this study, we used HRS as a discovery cohort and ELSA as an independent replication cohort to identify reproducible depressive symptom patterns in older adults. We aimed to derive harmonised CES-D-8 symptom-domain profiles, evaluate their cross-cohort structural replication, compare their baseline demographic, functional and health profiles, and test their associations with future elevated depressive symptom status, ADL worsening, IADL worsening and all-cause mortality. We further examined whether symptom patterns added prognostic information beyond the baseline CES-D total score, focusing on complementarity between symptom configuration and symptom severity rather than replacement of the total score.

## 2 Methods

### 2.1 Study design and data sources

**Figure 1:**
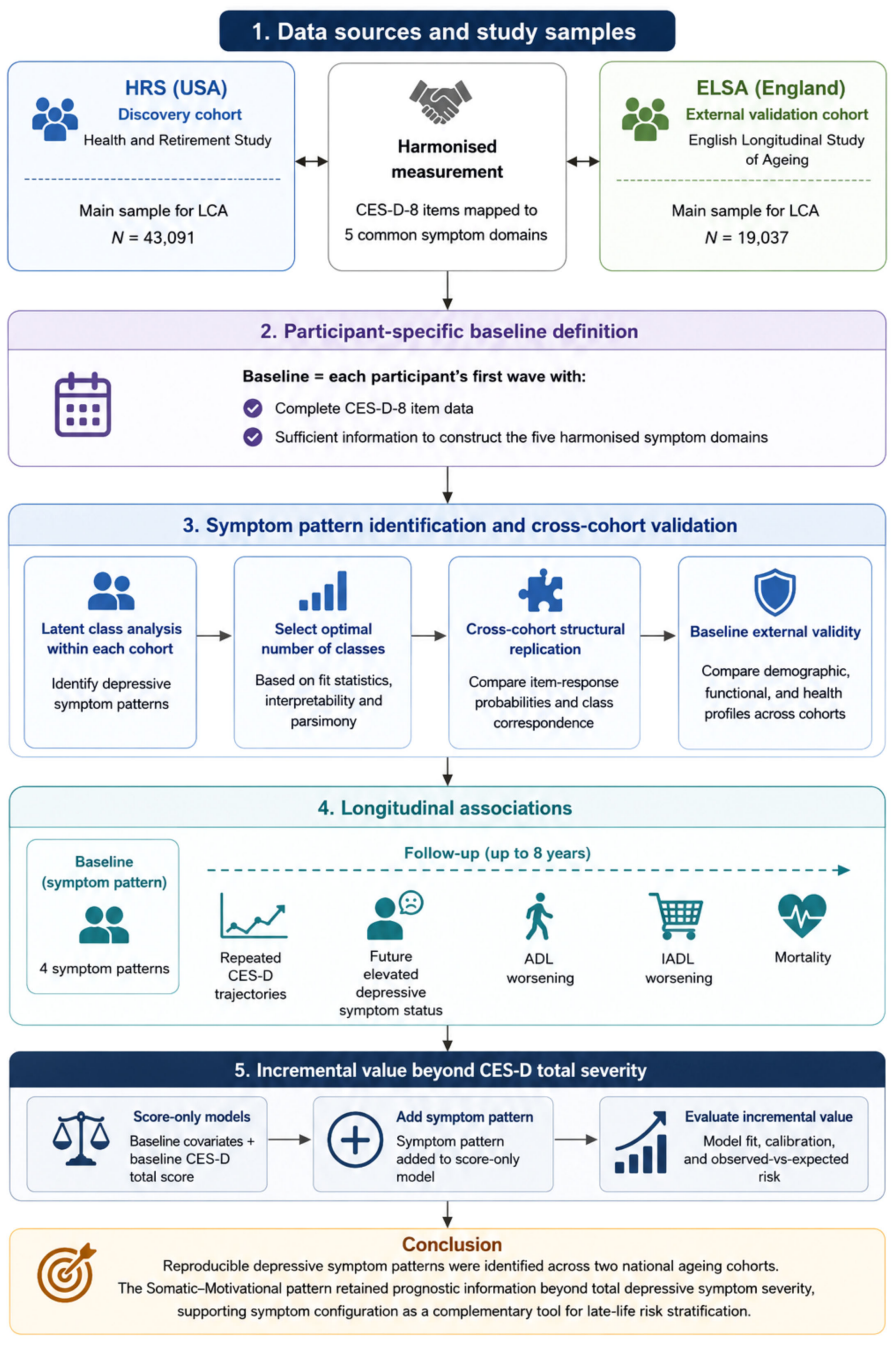
Study design and analytic workflow. The image summarises the cohort selection, baseline CES-D-8 symptom-domain construction, latent class analysis, cross-cohort structural replication between HRS and ELSA, baseline external-validity profiling, and longitudinal outcome analyses for depressive symptoms, ADL/IADL worsening and mortality.

We conducted a harmonised longitudinal cohort analysis to identify reproducible depressive symptom patterns in older adults and to examine their cross-cohort reproducibility and prospective associations with later outcomes. The Health and Retirement Study (HRS) in the United States served as the discovery cohort, and the English Longitudinal Study of Ageing (ELSA) in England served as the external replication cohort. HRS is a nationally representative longitudinal study of US adults aged over 50 years and their spouses, designed to collect interdisciplinary information on ageing, health, socioeconomic circumstances, family structure, work, retirement and health-care use (19). ELSA is a nationally representative longitudinal study of adults aged 50 years and older in England, developed to provide comparable ageing-related information on health, functioning, cognition, economic position, social participation and mortality (20). The two cohorts have similar ageing-research aims and overlapping CES-D-8 depressive symptom measures, which made them suitable for harmonised cross-cohort analysis. The study design and analytic workflow are shown in (**Figure 1**).

### 2.2 Study waves, baseline definition and analytic samples

HRS waves 2 to 16 and ELSA waves 1 to 9 were included. Item-level completeness varied across participants and waves, so baseline was defined separately for each participant as the first wave with complete CES-D-8 item data and sufficient information to construct the harmonised symptom-domain indicators. This definition used each participant’s earliest eligible observation and kept the symptom inputs complete and comparable for latent class analysis.

The primary analysis included the full eligible older-adult sample. Baseline CES-D total score, including the commonly used CES-D >=3 threshold, was not used as an inclusion criterion for the main latent class analysis (10). This population-based sampling strategy allowed symptom-pattern heterogeneity to be estimated across the broader older population before testing whether the resulting patterns stratified later health risks.

Using this definition, 43,091 HRS participants and 19,037 ELSA participants entered the main latent class analysis. Longitudinal outcome analyses were subsequently constructed within these baseline LCA samples according to the availability of each follow-up outcome.

### 2.3 Harmonisation of depressive symptom domains

Depressive symptoms were measured with the eight-item Center for Epidemiologic Studies Depression scale (CES-D-8) in both cohorts (10, 11). The item content was used to construct harmonised symptom-domain indicators that captured symptom configuration in addition to overall severity. Domain construction was guided by the multidimensional structure of CES-D symptoms and by the need to use items with comparable wording and coding across HRS and ELSA (11, 12).

Five binary symptom domains were derived for the main analysis. The negative affect domain was coded positive if either feeling depressed or feeling sad was endorsed. The anhedonia domain was coded from the two positive-affect items, feeling happy and enjoying life, after reverse coding. The fatigue/motivation domain was coded positive if either everything was an effort or could not get going was endorsed. Sleep disturbance was based on restless sleep, and loneliness/social distress was based on feeling lonely. For two-item domains, endorsement of either item was sufficient to define the domain as positive; for one-item domains, the binary item itself defined the domain.

This domain-level harmonisation retained the major affective, motivational, sleep and social components represented in the CES-D-8 while providing a more parsimonious structure for cross-cohort comparison. This approach also limited repeated weighting of closely related item pairs, including depressed/sad, happy/enjoying life and effort/could not get going, in the latent class model. Domain-level indicators reduced dependence on single-item response distributions and improved comparability between HRS and ELSA. To evaluate this harmonisation strategy, we examined within-domain correlations for paired CES-D-8 items and repeated the LCA using the original eight CES-D items as separate indicators. Variables with less consistent availability across cohorts or waves, including pain and broader physical, cognitive or psychosocial measures, were reserved for external-validity or extended-profile analyses and were not included in the main latent class model.

### 2.4 Latent class modelling

Latent class analysis (LCA) was used to identify subgroups of older adults with similar configurations of depressive symptoms (21). The five harmonised binary symptom domains were entered as observed indicators. All LCA models were fitted in R using a custom finite-mixture Bernoulli EM algorithm. Models with 1 to 6 classes were fitted separately in HRS and ELSA, with 80 random starts, a maximum of 500 EM iterations and a relative log-likelihood convergence tolerance of 1 × 10−7. For each class number, the solution with the highest log-likelihood across random starts was retained to reduce the risk of local maxima. The primary model assumed conditional independence between the five symptom-domain indicators; residual associations or local dependence terms were not explicitly modelled. We therefore assessed within-domain item correlations and performed an item-level CES-D-8 LCA sensitivity analysis to examine whether the domain-level solution was robust. In keeping with the aim of estimating symptom patterns rather than a unidimensional severity scale, the LCA model used the joint pattern of domain endorsement to assign individuals probabilistically to latent classes.

Models with 1 to 6 classes were fitted separately in HRS and ELSA. Class enumeration considered the Bayesian information criterion (BIC), sample-size adjusted BIC, entropy, mean average posterior probability, minimum class size, interpretability of the item-response probability profiles, split-half stability within HRS and structural replication in ELSA (22, 23). Because the 3-class solution was more parsimonious but the 4-class solution showed better likelihood-based fit, we specifically compared the 3-class and 4-class models with respect to symptom-profile separation, class size, interpretability and cross-cohort reproducibility. A 4-class solution was selected as the main model.

After model fitting, class labels were assigned by inspecting the item-response probabilities for the five domains rather than by relying on the arbitrary numeric order of the latent classes. The four classes were labelled Minimal Symptoms, Somatic-Motivational, Affective-Social and Global Distress.

### 2.5 Cross-cohort structural replication

Structural replication was assessed after fitting the five-domain LCA models independently in both cohorts. Because the ELSA model was independently estimated rather than obtained by directly applying fixed HRS model parameters, this analysis was interpreted as cross-cohort structural replication rather than formal validation of an HRS-derived model. In ELSA, as in HRS, models with 1 to 6 classes were estimated, and the 4-class solution was selected by BIC and sample-size adjusted BIC. We then compared the HRS 4-class solution with the corresponding ELSA 4-class solution to test whether the main symptom configurations identified in the discovery cohort were recovered in an independent ageing cohort.

Class correspondence was determined from the item-response probability profiles across negative affect, anhedonia, fatigue/motivation, sleep disturbance and loneliness/social distress. Numeric class labels from the LCA output were treated as arbitrary. We therefore matched classes by the shape of the five-domain probability profile and then applied the prespecified descriptive labels.

For each matched HRS-ELSA class pair, we quantified structural similarity using the Pearson correlation of item-response probabilities, root mean squared error, mean absolute difference and Jensen-Shannon divergence. Correlation described whether the relative symptom pattern was preserved across cohorts. RMSE and mean absolute difference described the absolute size of probability differences. Jensen-Shannon divergence provided a distributional measure of similarity (24). A full 4 by 4 pairwise similarity matrix was also calculated to check whether the matched classes were more similar to their same-label counterparts than to alternative classes.

### 2.6 Baseline external-validity analysis

Baseline external validity was assessed by asking whether classes with similar symptom profiles also showed similar demographic, functional and health profiles in HRS and ELSA. For each cohort, we summarised baseline characteristics by latent class. The variables included age, sex, education, marital status, body mass index, chronic disease count, ADL count, IADL count, cognitive score, current smoking and current drinking. The main standardised external-validity analysis focused on variables available in the preferred baseline analysis dataset. Self-rated health and poor/fair self-rated health were subsequently incorporated into the extended descriptive profiling analysis using available cohort-specific sources.

Raw baseline levels were expected to differ between the two cohorts because of differences in population structure, measurement scales and survey context. We therefore compared class profiles using within-cohort standardised deviations. For each baseline variable, the cohort-wide mean and standard deviation were calculated, and each class-specific mean or proportion was expressed as a deviation from the cohort mean divided by the cohort standard deviation. For cognition, higher values indicated better performance; because the HRS and ELSA cognitive scales differed, only within-cohort standardised deviations were compared.

Same-label class similarity was then quantified by comparing the standardised HRS and ELSA profiles for each class. We calculated Pearson correlation, Spearman rank correlation, root mean squared error, mean absolute difference and sign concordance. Pearson and Spearman correlations described the similarity of the overall profile shape and ranking. RMSE and mean absolute difference described the magnitude of cross-cohort profile differences. Sign concordance indicated whether class deviations from the cohort mean pointed in the same direction across cohorts. A full 4 by 4 cross-class similarity matrix was also calculated to assess whether each HRS class most closely resembled its same-label ELSA class.

### 2.7 Extended descriptive profiling

We performed an extended descriptive profiling analysis to characterise the clinical context of the four symptom patterns beyond the variables used for the main standardised external-validity analysis. This analysis used the fixed 4-class solution and summarised the same extended variable set across all classes in both cohorts.

The extended profile included sleep disturbance, pain, physical activity proxies, body mass index, chronic disease indicators, frailty-related descriptive components, loneliness, self-rated health and social isolation proxies. Continuous variables were summarised as mean with standard deviation, and binary variables were summarised as counts and percentages.

The purpose of this analysis was descriptive. It was used to clarify whether the symptom patterns corresponded to broader physical, functional or social profiles, and to provide clinical context for the longitudinal analyses. It was not used to redefine the latent classes, which remained based on the five harmonised CES-D-derived symptom domains.

### 2.8 Longitudinal outcomes

Longitudinal analyses began after each participant’s baseline wave. We examined five follow-up outcomes: repeated CES-D total score, future elevated depressive symptom status, ADL worsening, IADL worsening and all-cause mortality. Outcome-specific analysis samples were defined according to the availability of follow-up measurements.

Follow-up CES-D total score was analysed as a repeated continuous outcome using post-baseline waves with complete CES-D-8 item data. This analysis described subsequent symptom trajectories by baseline symptom pattern. Future elevated depressive symptom status was defined at each post-baseline wave as a CES-D-8 total score >=3. This person-wave outcome captured whether participants were above the elevated-symptom threshold during follow-up, regardless of whether their score had increased or decreased from baseline.

ADL worsening and IADL worsening were defined at the participant level. ADL worsening was coded when ADL count at any future wave exceeded the participant’s baseline ADL count. IADL worsening was defined in the same way using IADL count. All-cause mortality was analysed as time from baseline to death or censoring, with participants censored at the last wave with available follow-up information if no death was recorded. Death was therefore modelled as a separate survival outcome rather than treated only as loss to follow-up from repeated symptom or functional assessments.

### 2.9 Longitudinal statistical models

Longitudinal models were chosen according to the structure of each outcome. Follow-up CES-D total score was analysed with a linear mixed model including a participant-level random intercept (25). The model included baseline symptom pattern, time since baseline and a symptom pattern by time interaction. The interaction term tested whether subsequent CES-D trajectories differed by baseline symptom pattern. The model adjusted for age, sex, education, marital status, baseline wave and baseline CES-D total. Adjusted predicted CES-D total scores were estimated at 2, 4, 6 and 8 years after baseline by marginalising over the empirical covariate distribution within each cohort.

Future elevated depressive symptom status was analysed with pooled logistic regression at the person-wave level (26). Models included follow-up time terms, and participant-clustered robust standard errors were used to account for repeated observations from the same participant (27). ADL worsening and IADL worsening were analysed with participant-level logistic regression. All logistic models used Class 1, the Minimal Symptoms pattern, as the reference category and reported odds ratios with 95% confidence intervals.

For the logistic outcomes, we used a staged adjustment strategy. Model 0 included symptom pattern only. Model 1 adjusted for age, sex, education, marital status and baseline wave; the future elevated depressive symptom status model additionally included follow-up time, and the ADL and IADL models additionally included the corresponding baseline functional count and number of future auxiliary waves. Model 2 added baseline CES-D total and served as the primary model for assessing whether symptom patterns provided prognostic information beyond total depressive symptom severity. Model 3 further adjusted for current smoking, current drinking, chronic disease count and baseline functional status, with outcome-specific handling of baseline ADL and IADL to avoid adjusting for the same baseline function twice.

All-cause mortality was analysed with Cox proportional hazards models using time since baseline as the time scale (28). Participants without a recorded death were censored at the last wave with available follow-up information. The primary Cox model adjusted for age, sex, education, marital status, baseline wave and baseline CES-D total. The robustness Cox model additionally adjusted for current smoking, current drinking, chronic disease count, baseline ADL count and baseline IADL count. Hazard ratios and 95% confidence intervals were reported.

As a classification-confidence sensitivity analysis, we repeated the main future elevated depressive symptom, ADL worsening, IADL worsening and mortality models after restricting the sample to participants with maximum posterior class-assignment probabilities of at least 0.60 and, more stringently, at least 0.70. These thresholds were used to evaluate whether the main longitudinal associations were robust among participants with moderate and higher certainty of latent class assignment. The 0.60 threshold was chosen to retain a sufficient number of participants while reducing the influence of highly uncertain assignments, whereas the 0.70 threshold provided a stricter assessment of classification confidence.

### 2.10 Incremental value beyond CES-D total score

We examined whether depressive symptom patterns added information beyond baseline CES-D total score. This analysis was designed to test complementarity between symptom severity and symptom configuration. It did not assume that symptom pattern should replace the CES-D total score.

For follow-up CES-D trajectories, we compared three linear mixed models: a score-only trajectory model including baseline CES-D total and its interaction with time, a profile-only trajectory model including symptom pattern and its interaction with time, and a score-plus-profile trajectory model including both baseline CES-D total and symptom pattern with their time interactions. The main comparison was the score-plus-profile model versus the score-only model.

For future elevated depressive symptom status, ADL worsening, IADL worsening and all-cause mortality, we fitted three risk models within each cohort. Model A included baseline covariates and baseline CES-D total. Model B included baseline covariates and symptom pattern. Model C included baseline covariates, baseline CES-D total and symptom pattern. For the binary outcomes, model performance was summarised using Brier score, AIC, BIC, log-likelihood and calibration intercept and slope (29). For mortality, 8-year mortality risk was used to calculate Brier score and calibration metrics (29). Likelihood-ratio tests were used for the nested comparison of Model C versus Model A; Model B versus Model A was treated as a non-nested comparison.

We also examined whether symptom patterns separated future risk among participants with similar baseline CES-D total scores. Baseline CES-D total was grouped as 0-1, 2-3, 4–5 and >=6, and also as <3 versus >=3. Within each score stratum, class-specific observed and adjusted risks were estimated where sample size and event counts were sufficient. Strata with sparse class-specific data were flagged as unstable.

Finally, we assessed observed versus expected risk using the score-only model as the expected-risk model. For each symptom pattern, observed risk, expected risk, observed-minus-expected difference and observed-to-expected ratio were summarised. A multi-outcome adjusted risk profile was then constructed from the score-plus-profile model to show class-specific adjusted risk differences versus Class 1 across future elevated depressive symptom status, ADL worsening, IADL worsening and mortality.

## 3 Results

### 3.1 Analytic samples

The main LCA sample included 43,091 HRS participants and 19,037 ELSA participants with complete CES-D-8 item data at their first eligible baseline wave and sufficient information to construct the five harmonised symptom domains. HRS contributed baseline observations from waves 2 to 16, and ELSA contributed baseline observations from waves 1 to 9. These samples formed the denominator for symptom-pattern derivation in HRS and structural replication in ELSA.

Follow-up analyses were built within these baseline LCA samples and varied by outcome availability. In the primary adjusted person-wave model for future elevated depressive symptom status, 36,366 HRS participants contributed 217,583 follow-up observations, and 13,436 ELSA participants contributed 60,505 follow-up observations. Participant-level ADL and IADL worsening analyses included 36,882 and 36,876 HRS participants, respectively, and 13,686 ELSA participants for both outcomes. The mortality models included 40,080 HRS participants and 16,294 ELSA participants.

Detailed outcome-specific sample flow is shown in **Supplementary Table 10**. In the primary adjusted models, complete-case exclusions were minimal in HRS and were mainly due to missing education or marital-status information; in ELSA, exclusions were larger and were mainly due to missing education information. Baseline characteristics of included and excluded participants are shown in **Supplementary Table 11**.

### 3.2 Latent class solution and symptom patterns

For the five-domain model in HRS, the 4-class solution was selected by BIC and sample-size adjusted BIC. The 4-class model had a BIC of 201,669.8, sample-size adjusted BIC of 201,596.7, entropy of 0.661 and mean average posterior probability of 0.736. The smallest class represented 10.9% of the HRS sample. The 4-class solution was retained because it showed lower BIC and sample-size adjusted BIC than the 3-class solution in both HRS and ELSA. In addition, whereas the 3-class model identified only a single active symptom group, the 4-class model further distinguished two clinically meaningful and reproducible active profiles—Somatic-Motivational and Affective-Social. This additional differentiation improved clinical interpretability and was relevant for subsequent longitudinal analyses, as the two profiles exhibited distinct external and prognostic patterns. Therefore, the 4-class solution provided a better balance of model fit and clinical relevance. Model-fit indices for the 1- to 6-class solutions are shown in (**Table 1**).

**Table 1:** Model-fit indices for 1–6 class solutions in HRS and ELSA.

Cohort	Classes	Participants	BIC	aBIC	Entropy	Mean APP	Smallest class (%)	Selected main model
HRS	1	43091	236521.179	236505.289		1.0	100.0	
HRS	2	43091	203133.538	203098.58	0.84	0.943	23.26	
HRS	3	43091	202166.21	202112.184	0.589	0.815	15.386	
HRS	4	43091	201669.815	201596.721	0.661	0.736	10.868	Yes
HRS	5	43091	201719.955	201627.793	0.64	0.68	4.839	
HRS	6	43091	201782.662	201671.432	0.539	0.537	2.151	
ELSA	1	19037	101297.324	101281.435		1.0	100.0	
ELSA	2	19037	88067.668	88032.71	0.799	0.94	22.971	
ELSA	3	19037	87462.642	87408.617	0.623	0.829	12.985	
ELSA	4	19037	87372.164	87299.072	0.645	0.745	6.062	Yes
ELSA	5	19037	87425.279	87333.119	0.691	0.745	0.704	
ELSA	6	19037	87479.635	87368.407	0.549	0.613	3.204	

**Figure 2:**
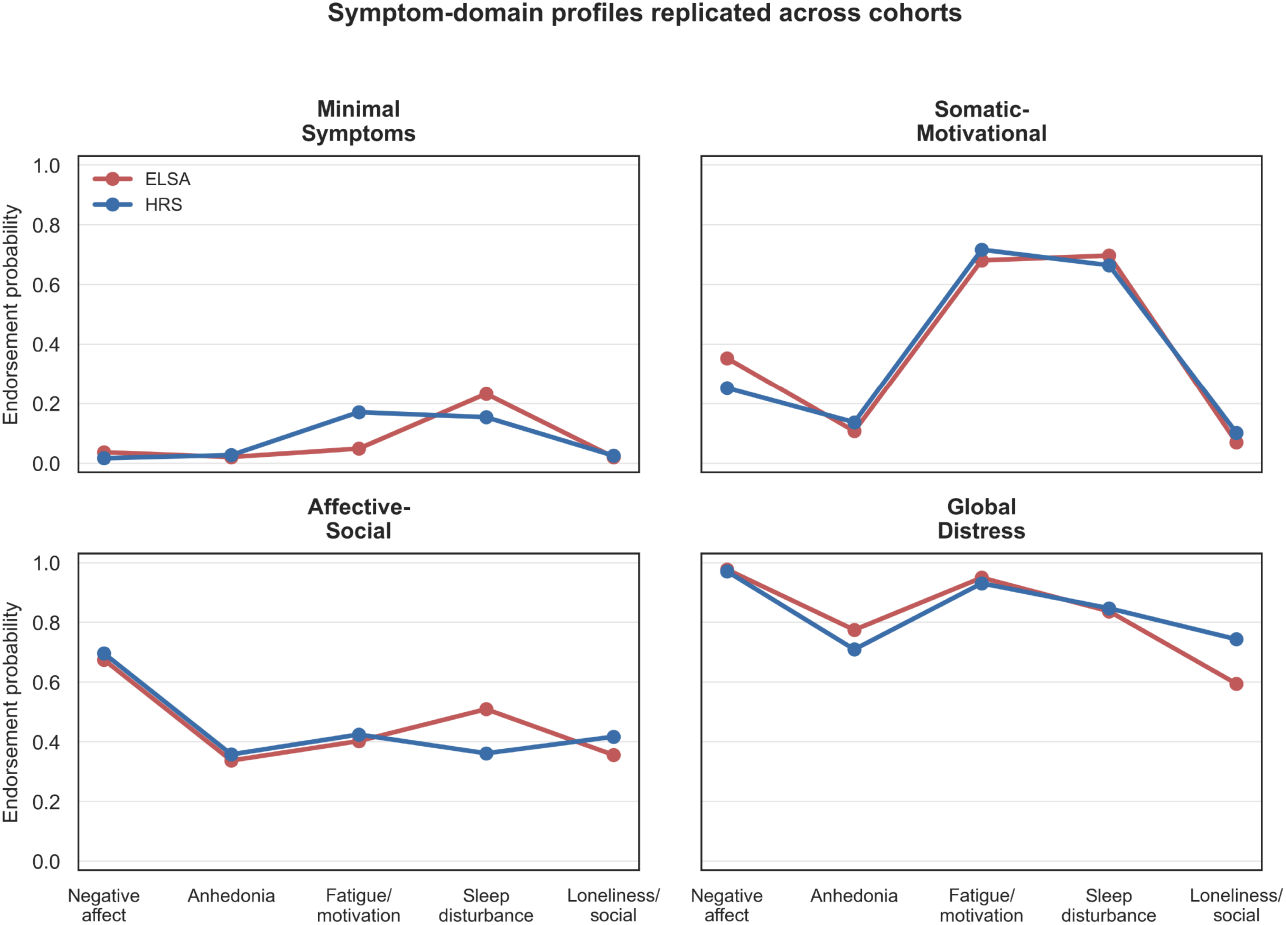
Symptom-domain profiles replicated across cohorts.

The four classes had clinically interpretable symptom profiles in the discovery cohort. Class 1, Minimal Symptoms, was the largest group. Class 2, Somatic-Motivational, was marked by high fatigue/motivation and sleep disturbance probabilities with lower affective and loneliness probabilities. Class 3, Affective-Social, was characterised mainly by negative affect and loneliness/social distress. Class 4, Global Distress, had high endorsement probabilities across most or all symptom domains. Symptom-domain profiles in HRS and ELSA are shown in (**Figure 2**).

Additional sensitivity analyses supported the domain-level symptom modelling strategy. The paired CES-D-8 items used to construct the two-item domains showed moderate within-domain correlations in both cohorts (**Supplementary Table 7**). These results indicate that the paired items captured related symptom content, supporting their grouping into broader symptom domains together with their conceptual similarity. In the exploratory item-level LCA using the original eight CES-D items, the 4-class solution recovered profiles corresponding to the main domain-based patterns. Minimal Symptoms, Somatic-Motivational, Affective-Social and Global Distress accounted for 69.2%, 12.8%, 8.5% and 9.4% of HRS participants, and 67.7%, 17.2%, 6.4% and 8.7% of ELSA participants, respectively (**Supplementary Figure 5**). Model-fit indices for the original-item LCA models are shown in **Supplementary Table 8**. These findings support the robustness of the main symptom-profile interpretation based on the five-domain model.

### 3.3 Cross-cohort structural replication

The 4-class structure showed cross-cohort replication at the symptom-profile level. The matched Somatic-Motivational classes were highly similar across HRS and ELSA (item-probability correlation, 0.980; RMSE, 0.054). The Affective-Social and Global Distress patterns also showed good structural correspondence, with correlations of 0.828 and 0.860, respectively. The Minimal Symptoms pattern had a lower correlation (0.631), largely because absolute endorsement probabilities were close to the lower bound for several domains, but absolute differences remained small (MAD, 0.046). These results support a reproducible 4-pattern structure in the independent ELSA cohort.

### 3.4 Baseline external validity and extended profiles

**Figure 3:**
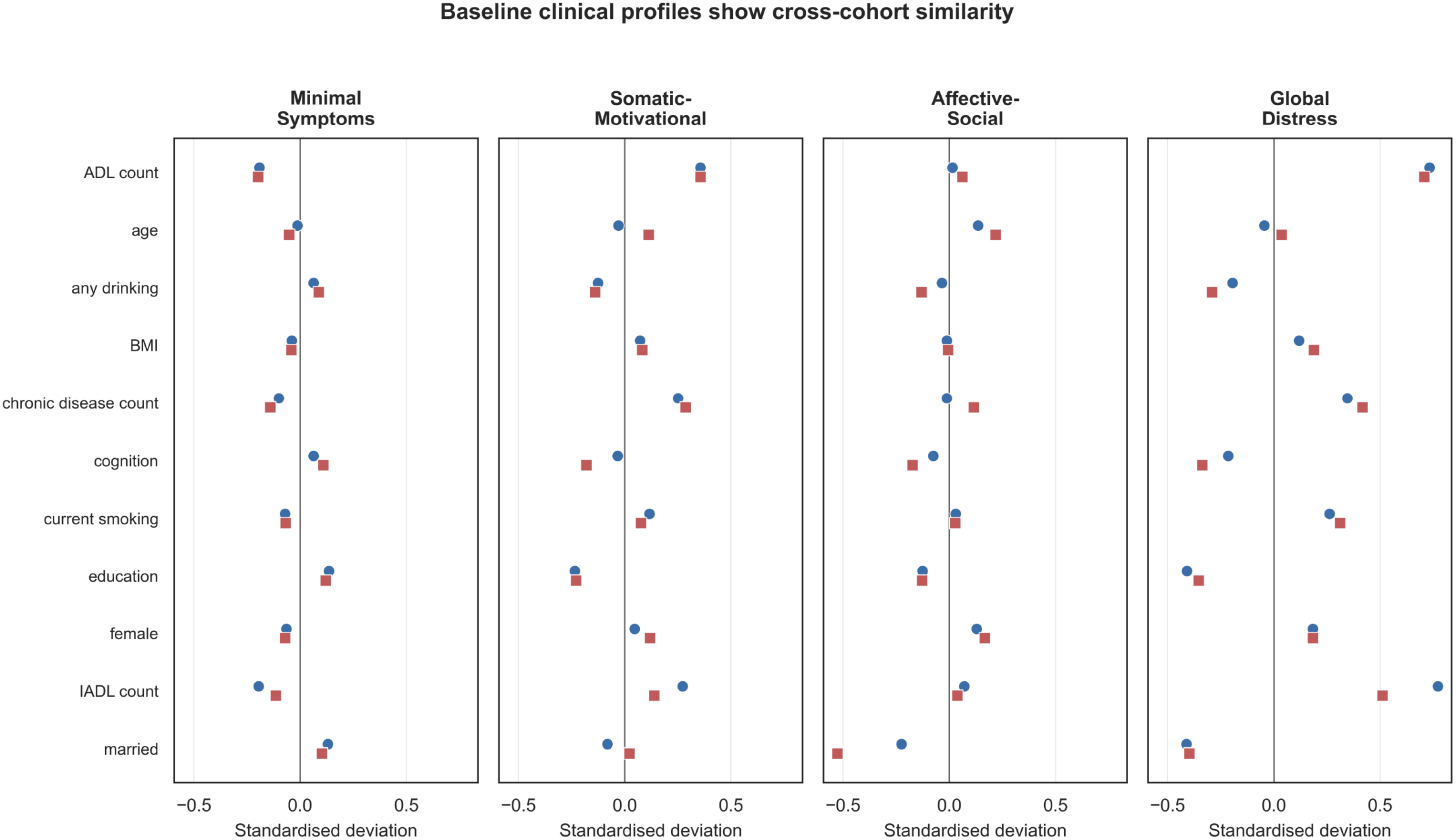
Baseline clinical profiles show cross-cohort similarity.

Standardised baseline profiles also supported cross-cohort similarity in external clinical profiles. Same-label HRS and ELSA classes showed high overall profile similarity across 11 baseline demographic, health and functional variables. Pearson correlations ranged from 0.882 for the Somatic-Motivational pattern to 0.966 for the Global Distress pattern. The Minimal Symptoms and Global Distress patterns showed the strongest direction concordance, while the Somatic-Motivational and Affective-Social patterns showed somewhat lower sign concordance but retained similar overall profile shapes. Baseline characteristics by latent class and cohort are summarized in (**Table 2**), and baseline clinical profiles by cohort are shown in (**Figure 3**).

**Table 2:** Baseline characteristics by latent class and cohort.

Characteristic	HRS_Minimal	HRS_Somatic	HRS_Affective	HRS_Global	ELSA_Minimal	ELSA_Somatic	ELSA_Affective	ELSA_Global
ADL count	0.09 ± 0.46	0.54 ± 1.14	0.26 ± 0.80	0.84 ± 1.42	0.16 ± 0.58	0.67 ± 1.24	0.40 ± 0.95	0.99 ± 1.51
BMI	30.19 ± 6.31	30.92 ± 6.70	30.37 ± 6.46	31.22 ± 7.13	28.09 ± 4.98	28.74 ± 5.53	28.28 ± 5.60	29.31 ± 6.96
IADL count	0.15 ± 0.47	0.50 ± 0.92	0.35 ± 0.77	0.86 ± 1.23	0.02 ± 0.16	0.09 ± 0.37	0.06 ± 0.26	0.19 ± 0.54
age	60.16 ± 10.32	59.99 ± 10.84	61.73 ± 11.46	59.82 ± 10.94	60.36 ± 10.04	62.08 ± 11.08	63.18 ± 11.76	61.28 ± 11.14
any drinking	17548 (62.7%)	2500 (53.4%)	3098 (57.9%)	2526 (50.0%)	11093 (91.8%)	2376 (84.8%)	911 (85.1%)	1422 (80.1%)
chronic disease count	0.36 ± 0.68	0.63 ± 0.92	0.43 ± 0.76	0.70 ± 1.01	0.74 ± 0.87	1.15 ± 1.07	0.99 ± 0.97	1.28 ± 1.11
cognition	3.79 ± 0.51	3.74 ± 0.58	3.71 ± 0.62	3.64 ± 0.72	10.50 ± 3.47	9.46 ± 3.59	9.48 ± 3.86	8.90 ± 3.78
current smoking	4661 (17.8%)	1100 (25.4%)	1086 (21.9%)	1454 (31.3%)	2012 (15.6%)	639 (21.2%)	223 (19.3%)	601 (30.3%)
education	2.04 ± 0.65	1.79 ± 0.63	1.87 ± 0.67	1.68 ± 0.63	1.89 ± 0.70	1.64 ± 0.67	1.71 ± 0.70	1.56 ± 0.65
female	15002 (53.6%)	2767 (59.1%)	3382 (63.2%)	3326 (65.9%)	6640 (51.5%)	1838 (61.0%)	731 (63.3%)	1273 (64.2%)
married	19569 (69.9%)	2793 (59.7%)	2821 (52.8%)	2209 (43.8%)	9465 (73.5%)	2101 (69.7%)	510 (44.2%)	996 (50.2%)

The extended descriptive profiles gave clinical context to the symptom patterns. The Minimal Symptoms pattern had the lowest overall burden across sleep, pain, physical activity, loneliness/social isolation and frailty-related descriptive components. The Somatic-Motivational pattern showed a more prominent physical and functional burden, including higher sleep disturbance, exhaustion-related features, pain, lower physical activity and chronic disease burden. The Affective-Social pattern was distinguished most clearly by loneliness and social isolation, with intermediate physical and functional burden. The Global Distress pattern showed broad elevation across symptom, physical and frailty-related domains, consistent with its high item-response probabilities across the CES-D-derived symptom domains.

In the extended profiles, self-rated health and poor/fair self-rated health were also compared across classes and showed higher perceived health burden in the Somatic-Motivational and Global Distress patterns, particularly in Global Distress (**Supplementary Table 4**).

### 3.5 Longitudinal outcomes

**Figure 4:**
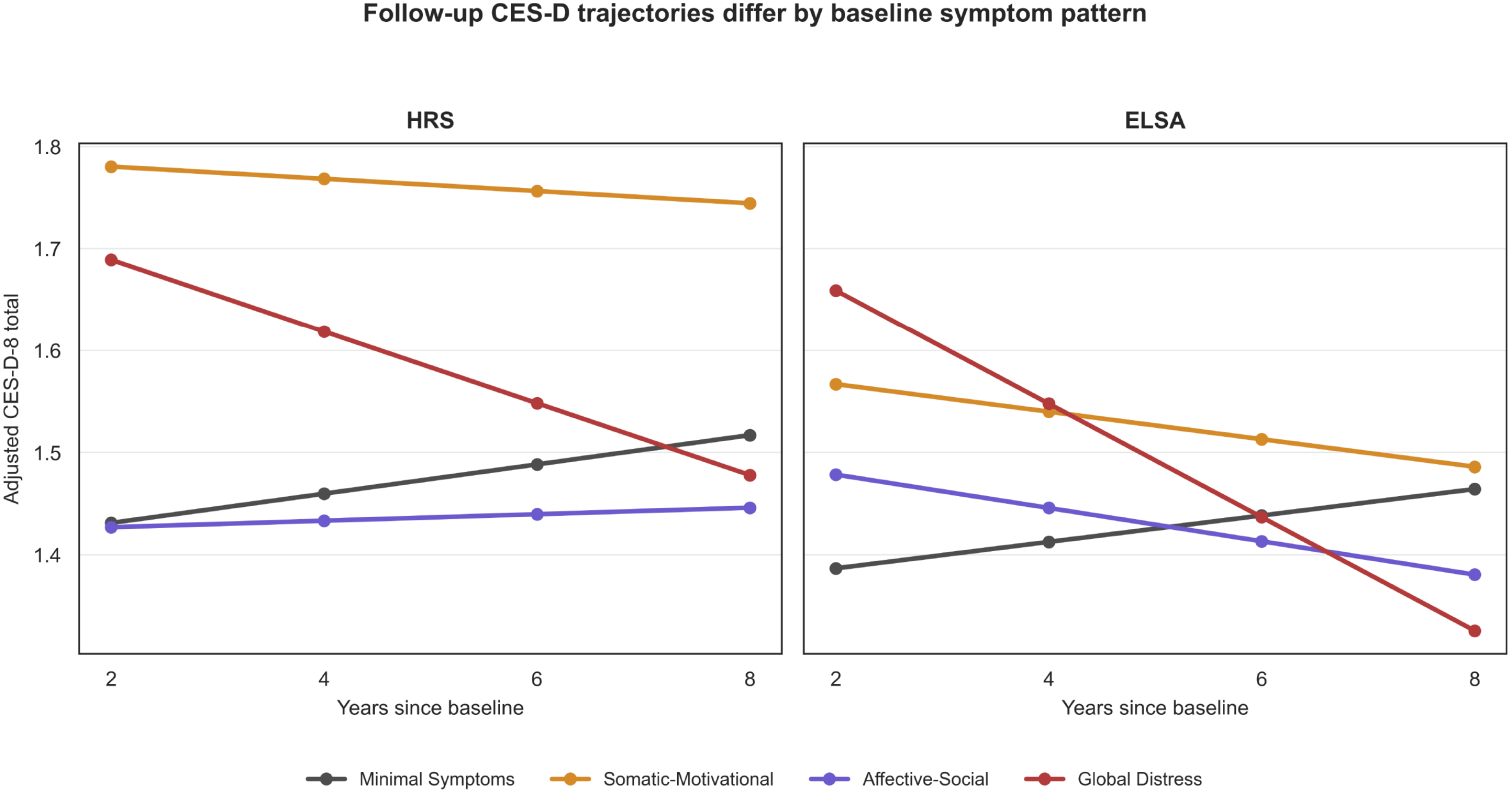
Points represent adjusted marginal mean CES-D total scores estimated at 2, 4, 6 and 8 years after baseline, with adjustment for age, sex, education, marital status, baseline wave and baseline CES-D total score. In HRS, adjusted CES-D values at 2, 4, 6 and 8 years were 1.47, 1.48, 1.50 and 1.51 for Minimal symptoms; 1.73, 1.74, 1.74 and 1.75 for somatic-motivational; 1.39, 1.41, 1.43 and 1.45 for affective-social; and 1.53, 1.51, 1.50 and 1.49 for global distress. In ELSA, the corresponding values were 1.45, 1.45, 1.44 and 1.44; 1.48, 1.49, 1.51 and 1.52; 1.36, 1.38, 1.40 and 1.43; and 1.36, 1.39, 1.42 and 1.45, respectively. Although class-by-time interactions were statistically significant in both cohorts, the absolute differences in adjusted CES-D values were modest and should be interpreted as differences in trajectory pattern rather than large differences in symptom burden.

Follow-up CES-D trajectories differed by baseline symptom pattern in both cohorts (class-by-time interaction: HRS, P < 0.001; ELSA, P < 0.001). The Global Distress pattern had the highest adjusted CES-D levels early in follow-up but declined over time. The Somatic-Motivational pattern showed more persistent follow-up symptom burden, particularly in HRS, where its adjusted CES-D total remained higher than the other non-Global Distress patterns through 8 years. The Minimal Symptoms pattern remained the lowest or among the lowest across follow-up, while the Affective-Social pattern showed a more modest trajectory after adjustment for baseline CES-D total. Adjusted follow-up CES-D trajectories are shown in (**Figure 4**).

In models that adjusted for baseline CES-D total (Model 2), the Somatic-Motivational pattern was the most consistent longitudinal risk pattern. It was associated with higher odds of future elevated depressive symptom status in both ELSA (OR, 1.39; 95% CI, 1.22-1.58) and HRS (OR, 1.53; 95% CI, 1.41-1.65). This association remained after further adjustment for smoking, drinking, chronic disease count and baseline functional status (Model 3).

**Figure 5:**
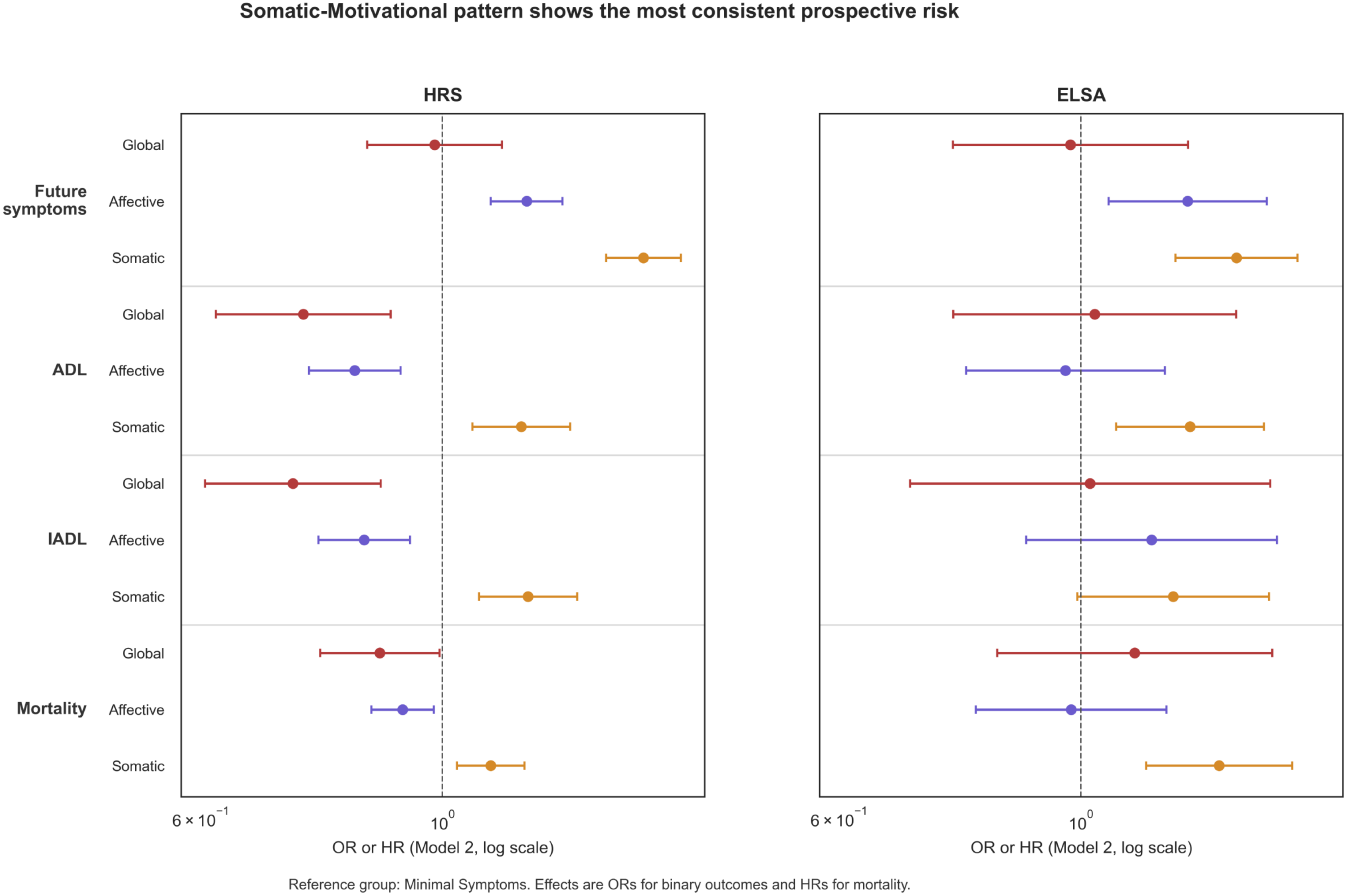
Somatic-motivational pattern shows the most consistent prospective risk.

For functional outcomes, the Somatic-Motivational pattern was associated with ADL worsening in both cohorts after adjustment for baseline CES-D total (ELSA: OR, 1.26; 95% CI, 1.08-1.47; HRS: OR, 1.18; 95% CI, 1.07-1.31). The association persisted in the robustness model. Evidence for IADL worsening was weaker across cohorts: the association was present in HRS but was not statistically stable in ELSA. The Somatic-Motivational pattern was also associated with higher all-cause mortality in both cohorts in the primary model (ELSA: HR, 1.34; 95% CI, 1.15-1.56; HRS: HR, 1.11; 95% CI, 1.03-1.19), with similar direction after further adjustment. Prospective associations with follow-up outcomes are shown in (**Figure 5**).

The Affective-Social pattern was associated with future elevated depressive symptom status in both cohorts after adjustment for baseline CES-D total, but it did not show a consistent excess risk for ADL worsening, IADL worsening or mortality. The Global Distress pattern had high baseline and early follow-up symptom burden, but its additional longitudinal risk was largely attenuated after baseline CES-D total was included. This pattern indicates that total symptom severity captured much of the future risk associated with the Global Distress profile, whereas the Somatic-Motivational pattern retained prognostic information beyond total score.

This conclusion was supported in the posterior-confidence sensitivity analysis. After restricting the sample to participants with maximum posterior probabilities of at least 0.60, the Somatic-Motivational pattern remained associated with higher risk of future elevated depressive symptom status, ADL worsening, IADL worsening and mortality in both cohorts in the primary adjusted models (**Supplementary Table 6**). At the more restrictive threshold of 0.70, the same direction of association remained evident for future elevated depressive symptom status in both cohorts, but the HRS Somatic-Motivational class became much smaller, limiting precision for the functional and mortality estimates.

### 3.6 Incremental value beyond CES-D total score

The profile-versus-score analyses showed that symptom patterns complemented, rather than replaced, the CES-D total score. In trajectory models, the score-plus-profile model improved model fit over the score-only model in HRS, while improvements in ELSA were smaller. The profile-only model generally did not outperform the score-only model, supporting the continued importance of total symptom severity.

For future risk outcomes, adding symptom pattern to the score-only model consistently improved likelihood-based fit. For future elevated depressive symptom status, the score-plus-profile model reduced AIC relative to the score-only model in both cohorts (ELSA, delta AIC = -152.3; HRS, delta AIC = -697.9), with statistically significant likelihood-ratio tests. Similar fit improvements were observed for ADL worsening, IADL worsening and mortality, supporting the incremental value of symptom configuration beyond total symptom severity.

To aid clinical interpretation, we additionally summarised adjusted absolute risks and risk differences from the score-plus-profile model (**Supplementary Table 12**). Compared with the Minimal Symptoms pattern, the Somatic-Motivational pattern showed higher adjusted risk of future elevated depressive symptoms in ELSA (24.2% vs 19.6%; risk difference, 4.6%, 95% CI 4.4–4.8) and HRS (27.5% vs 21.0%; risk difference, 6.5%, 95% CI 6.4–6.6). Corresponding risk differences were 4.5% and 3.4% for ADL worsening, 2.1% and 3.7% for IADL worsening, and 2.8% and 1.2% for mortality in ELSA and HRS, respectively.

**Figure 6:**
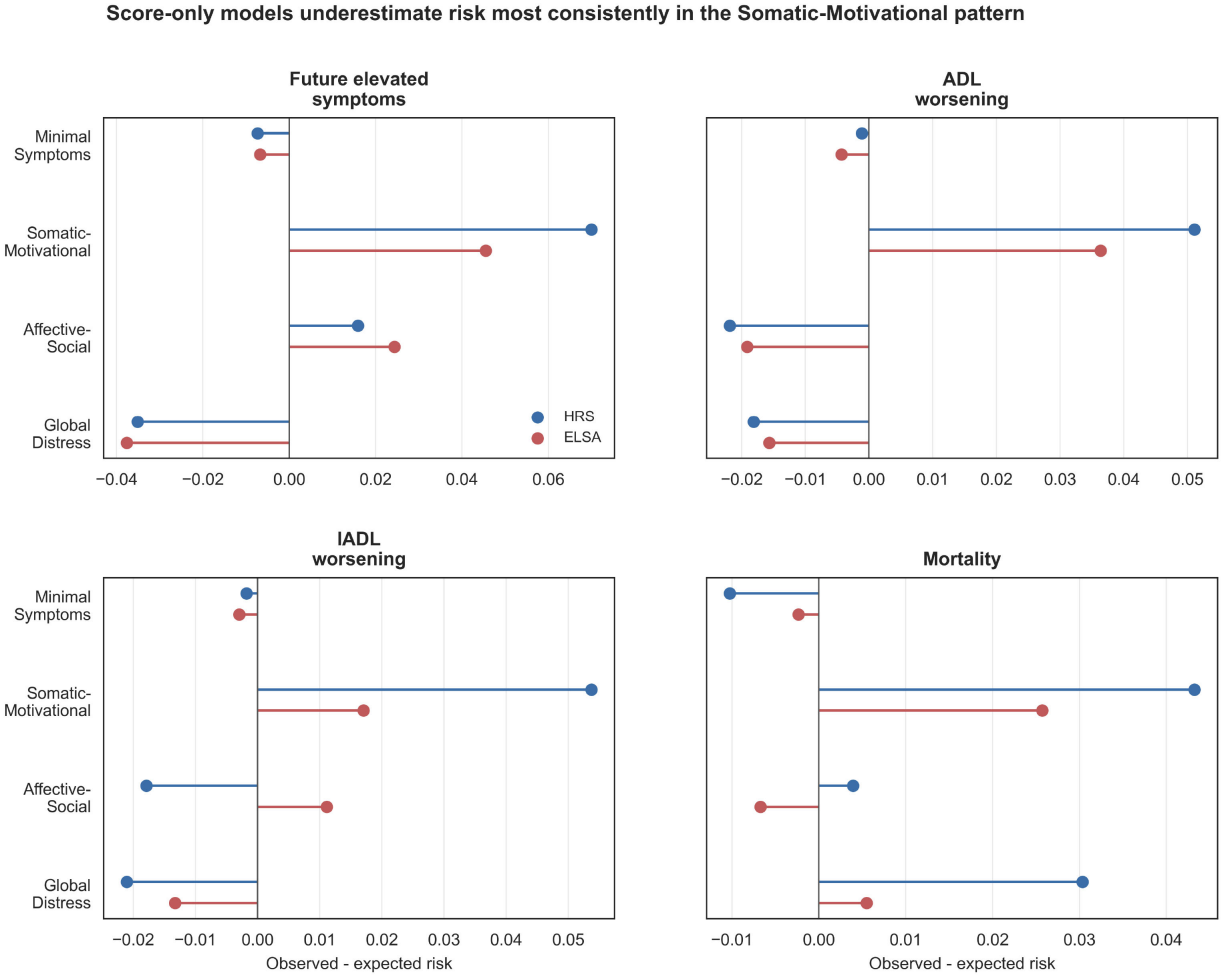
Score-only models underestimate risk most consistently in the somatic-motivational pattern.

Same-score-stratum and observed-versus-expected analyses identified the Somatic-Motivational pattern as the clearest source of incremental risk information. Within comparable CES-D total score strata, this pattern often showed higher future risk than other patterns, although some strata were limited by sample size and event counts. In observed-versus-expected analyses based on the score-only model, the Somatic-Motivational pattern had observed risks above expected risks for future elevated depressive symptom status, ADL worsening, IADL worsening and mortality in both cohorts. These findings suggest that CES-D total score alone may underestimate risk in this subgroup. Observed-minus-expected risk differences are shown in (**Figure 6**).

## 4 Discussion

In this harmonised analysis of two national ageing cohorts, we identified four depressive symptom patterns in both HRS and ELSA: Class 1, Minimal Symptoms; Class 2, Somatic-Motivational; Class 3, Affective-Social; and Class 4, Global Distress. These patterns showed cross-cohort structural replication and broadly similar baseline clinical profiles. Among the active symptom patterns, Class 2 was the most stable longitudinal risk pattern across depressive, functional and mortality outcomes. The overall findings indicate that symptom patterns should not replace the CES-D total score, but can provide complementary information beyond total symptom severity.

The cross-cohort reproducibility of the 4-pattern structure is central to the interpretation of this study. HRS and ELSA are drawn from different countries and differ in survey context, population composition and health-system setting, yet independently fitted models recovered clinically similar symptom configurations. This design moves the analysis beyond a single-cohort clustering exercise, an important concern because latent class solutions in depression can be sensitive to sample composition, indicator selection and model specification (30). The strongest structural correspondence was seen for the Somatic-Motivational, Affective-Social and Global Distress patterns, suggesting that these active profiles captured robust symptom-configuration signals rather than arbitrary data partitions. The lower correlation for the Minimal Symptoms pattern should be interpreted differently: when endorsement probabilities are uniformly low, small absolute differences can reduce correlation even when absolute probability differences remain minor. This distinction supports the cross-cohort reproducibility of the 4-pattern solution while also showing why both relative and absolute similarity metrics are needed when evaluating replication across cohorts.

The choice of a 4-class solution also reflects the balance between statistical fit, clinical interpretability and reproducibility. A 2-class solution would mainly separate lower-burden from higher-burden participants and would therefore provide little information beyond overall symptom severity. By contrast, the 4-class solution separated clinically distinct active profiles, especially Somatic-Motivational, Affective-Social and Global Distress, which differed not only in symptom configuration but also in longitudinal risk patterns. Although more complex solutions can sometimes improve selected model indices, the 5-class solution did not provide a comparably robust added pattern and instead introduced very small classes, especially in ELSA, raising concern about over-partitioning. The 4-class solution was therefore preferred because it jointly achieved the best BIC and sample-size adjusted BIC in both cohorts while also showing recovery of the same symptom-profile structure in an independent replication cohort.

Class 1, the Minimal Symptoms pattern, should be interpreted as the lowest symptom-probability profile in a population-based sample rather than as a clinically verified non-depressed group. Because the latent class analysis was conducted in the full eligible sample and did not require participants to meet a depressive-symptom threshold, this class represents older adults with low endorsement probabilities across the five CES-D-derived symptom domains. However, class membership is probabilistic and is not identical to a CES-D total-score category or a diagnostic status. Some individuals assigned to Minimal Symptoms may still have a CES-D total score at or above the commonly used threshold of 3, particularly if they endorsed several symptoms but remained closer to the low-probability profile than to the other latent patterns (31). Therefore, this class should not be used to rule out clinically relevant symptoms at the individual level. Its role in this study is to provide a low-burden reference pattern for comparing symptom configurations, while CES-D total score and clinical assessment remain necessary when evaluating individual symptom severity.

Class 2, the Somatic-Motivational pattern, was the most consistent longitudinal risk signal in this study. This group was not the class with the highest global symptom burden, but it showed the most consistent excess risk after adjustment for baseline CES-D total score. It predicted future elevated depressive symptom status in both cohorts and was also associated with ADL worsening and all-cause mortality. This suggests that a configuration dominated by fatigue, motivational difficulty and sleep disturbance may mark a broader state of health vulnerability in older adults. Importantly, this pattern should not be interpreted as a depression-specific subtype. In older adults, fatigue, motivational difficulty and sleep disturbance may also reflect frailty, chronic disease burden, pain, sleep problems, reduced activity or general somatic vulnerability, which may partly explain its associations with functional decline and mortality. Prior late-life depression research has linked somatic depressive symptoms with functional disability (32–34), and systematic reviews describe substantial overlap and bidirectional association between depression and frailty in later life (35, 36). This interpretation is also biologically and clinically plausible because exhaustion and low activity are core components of widely used frailty phenotypes (37, 38), and sleep problems in older adults have been associated with adverse health outcomes including mortality (39, 40). Our observational design cannot determine whether the Somatic-Motivational pattern causes later decline, but it indicates a subgroup for whom depressive symptoms, sleep, fatigue, activity limitation, chronic disease burden and functional vulnerability may warrant consideration in future studies of comprehensive follow-up assessment.

Class 3, the Affective-Social pattern, had a different interpretation. It was associated with future elevated depressive symptom status after adjustment for baseline CES-D total score, but did not show consistent additional risk for ADL worsening, IADL worsening or mortality. Its symptom profile and extended baseline characteristics suggest a pattern more closely tied to affective distress and loneliness/social distress than to broad physical decline. This fits longitudinal evidence that loneliness and social isolation are closely related to depressive symptoms in older adults (41–44), while their links with functional decline and mortality may depend on health status, social resources and follow-up context. Clinically, this pattern may indicate a need to assess emotional distress, loneliness and social connectedness during follow-up. These implications should be viewed as hypotheses for assessment and follow-up, not as evidence that symptom class membership determines a treatment pathway.

The attenuation of risk for Class 4, the Global Distress pattern, after adjustment for baseline CES-D total score is also informative. Global Distress was characterised by high endorsement across most or all symptom domains and showed the highest early follow-up CES-D levels. Because its defining feature is broad symptom burden, much of its future risk is expected to be captured by the total CES-D score. Therefore, the reduction in additional risk after total-score adjustment does not weaken the study argument; rather, it confirms that total symptom severity remains an important prognostic summary. The contrast between Global Distress and Somatic-Motivational clarifies the contribution of symptom configuration: the former reflects risk largely aligned with overall severity, whereas the latter retains prognostic information that is not fully represented by the total score.

The incremental-value analyses address the core scientific question of whether symptom configuration adds information beyond depressive symptom severity. The profile-only models generally did not replace score-only models, which supports the continued use of CES-D total score as a compact severity measure in ageing cohorts. However, adding symptom pattern to score-only models improved likelihood-based fit across outcomes, and observed-versus-expected analyses showed that the Somatic-Motivational pattern often had risks above those expected from total score alone. Same-score-stratum analyses pointed in the same direction: among older adults with similar CES-D total scores, future risk could differ by symptom configuration. These findings suggest a potential explanatory contribution of the phenotype approach. After screening or severity scoring, attention to the type of symptoms endorsed may help identify subgroups whose risk is underestimated by score-based assessment alone, but the modest changes in discrimination metrics indicate that practical prediction utility remains uncertain.

The posterior-confidence sensitivity analysis strengthens this interpretation. Restricting the analysis to participants with higher class-assignment certainty did not remove the main excess-risk signal for the Somatic-Motivational pattern at the >=0.60 threshold. When the threshold was increased to >=0.70, associations for future elevated depressive symptom status remained directionally similar in both cohorts, but the HRS Somatic-Motivational group became sparse, so attenuation of some ADL, IADL and mortality estimates is more consistent with reduced precision than with a clear loss of the underlying pattern.

These findings have pragmatic clinical and public health implications. In large ageing cohorts and primary-care-adjacent settings, the CES-D total score remains useful for rapid screening and severity summarisation. At the same time, a single total score may obscure whether symptoms are concentrated in somatic-motivational, affective-social or globally elevated domains. The Somatic-Motivational pattern may help generate hypotheses about older adults who could benefit from more comprehensive evaluation of depressive symptoms, sleep, fatigue, physical activity, ADL/IADL function and chronic disease burden. The Affective-Social pattern may highlight needs related to loneliness, social connection and emotional support. These patterns are best understood as risk-stratification and assessment aids rather than diagnostic categories, clinical subtypes or direct treatment rules.

This study has several strengths. It used two large, well-established national ageing cohorts and a discovery-replication design, with HRS used for discovery and ELSA used for independent structural replication. Depressive symptom indicators were harmonised across cohorts using comparable CES-D-8 content. The analysis went beyond latent class enumeration by testing symptom-profile replication, baseline external validity, extended clinical profiles and longitudinal outcomes. It also explicitly tested whether symptom patterns added information beyond CES-D total score across depressive, functional and mortality endpoints.

Several limitations should be considered. First, CES-D-8 is a brief screening measure of depressive symptom burden and cannot establish a clinical diagnosis of major depressive disorder. Second, the observational design precludes causal inference about symptom-pattern membership and later outcomes. Third, missing follow-up data and attrition may have introduced bias, particularly because participants with poorer health may have been less likely to contribute repeated depressive-symptom or functional measurements and more likely to die. Although mortality was analysed separately as a time-to-event outcome and ADL/IADL models accounted for the number of future auxiliary waves, we did not apply formal missing-data or informative-censoring approaches, such as multiple imputation, inverse-probability-of-censoring weighting or joint longitudinal-survival models. Fourth, although HRS and ELSA provide a strong cross-national replication setting, the generalisability of these findings beyond the United States and England, and beyond these cultural and measurement contexts, remains uncertain. Fifth, we did not formally test measurement invariance or impose equality constraints on item-response probabilities across cohorts; therefore, profile correlations and distance metrics support structural similarity but do not prove identical measurement of the latent classes. Finally, residual confounding remains possible because several relevant factors, including medication or antidepressant use, anxiety symptoms, detailed socioeconomic indicators, pain severity, physical activity, frailty, health-care access and specific multimorbidity patterns, were unavailable, inconsistently measured or examined only descriptively to preserve cross-cohort comparability and avoid additional complete-case loss or overadjustment.

Future research should test whether the same symptom patterns can be recovered in other countries, cultural contexts and clinical samples, ideally using both brief screening tools and more comprehensive depression assessments. Studies with clinical diagnostic information could clarify how these symptom patterns relate to depressive disorders, subthreshold depression and non-depressive health states. Latent transition or trajectory analyses could determine whether older adults move between Minimal Symptoms, Somatic-Motivational, Affective-Social and Global Distress patterns over time, and whether transitions predict changes in function or survival. Mechanistic work should examine why the Somatic-Motivational pattern carries excess risk, including sleep disturbance, inflammation, frailty, multimorbidity, polypharmacy and reduced activity as potential pathways. Intervention studies are ultimately needed to determine whether symptom-pattern-informed follow-up strategies improve outcomes.

## 5 Conclusion

In conclusion, depressive symptom patterns derived from harmonised CES-D-8 domains were reproducible across two national ageing cohorts and showed meaningful longitudinal associations. The Somatic-Motivational pattern was the clearest source of prognostic information beyond CES-D total score, whereas Global Distress primarily reflected high overall symptom severity and Affective-Social captured a more emotionally and socially oriented risk profile. These findings support symptom configuration as a complementary research construct for understanding late-life risk, alongside rather than instead of total depressive symptom severity; further work is needed before clinical risk-stratification use can be recommended.

## Data Availability

The original contributions presented in the study are included in the article/**Supplementary Material**. Further inquiries can be directed to the corresponding author.
