# Supplementary figures and images for: Depressive symptom patterns in older adults: evidence from two national ageing cohorts
Source: Front Psychiatry. 2026 Jul 10;17:1893371. doi: 10.3389/fpsyt.2026.1893371 (PMC13397582; doi:10.3389/fpsyt.2026.1893371)

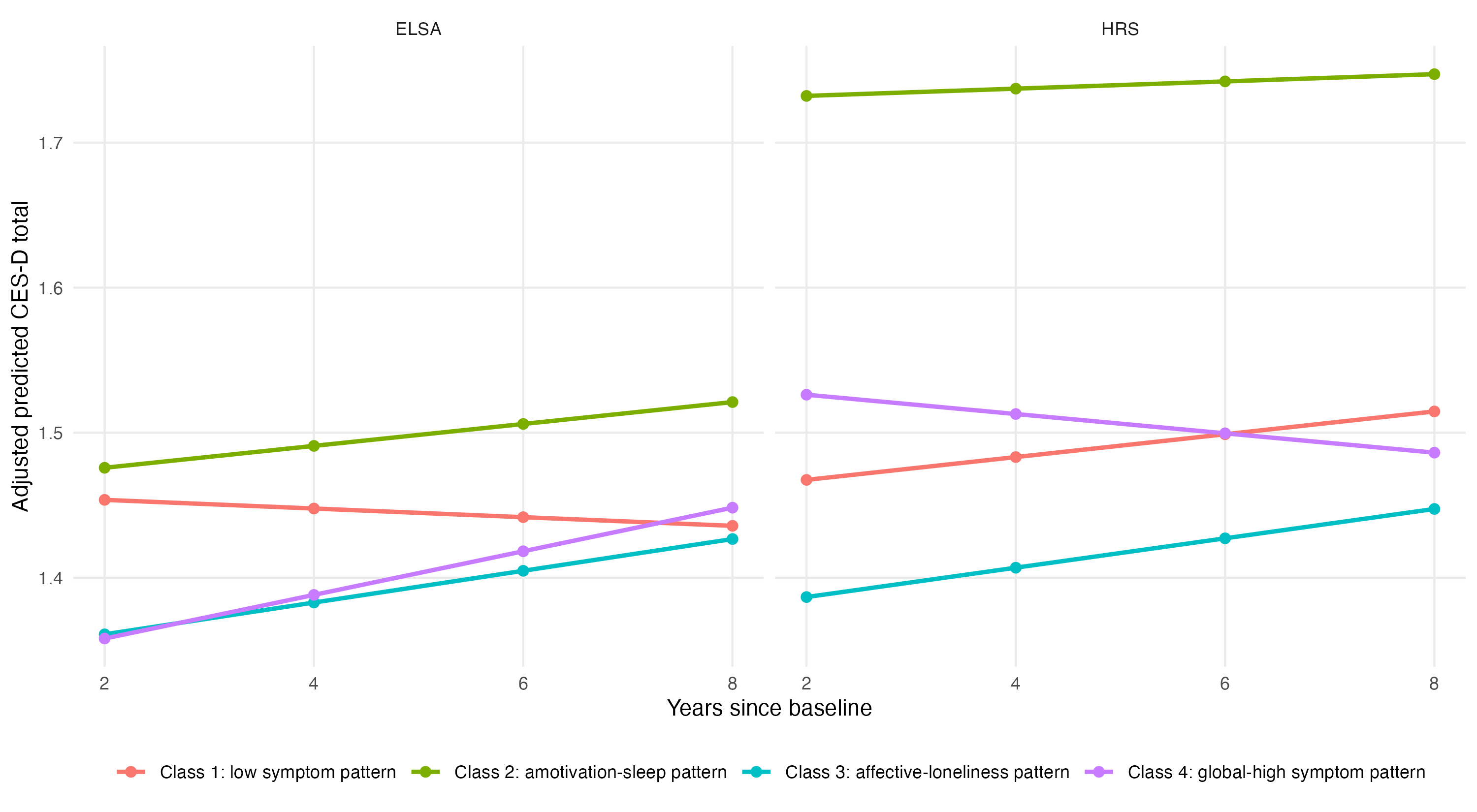

Supplement: Supplementary file 1 [file DataSheet1.zip › supplementary_figures/supplementary_figure4.png]

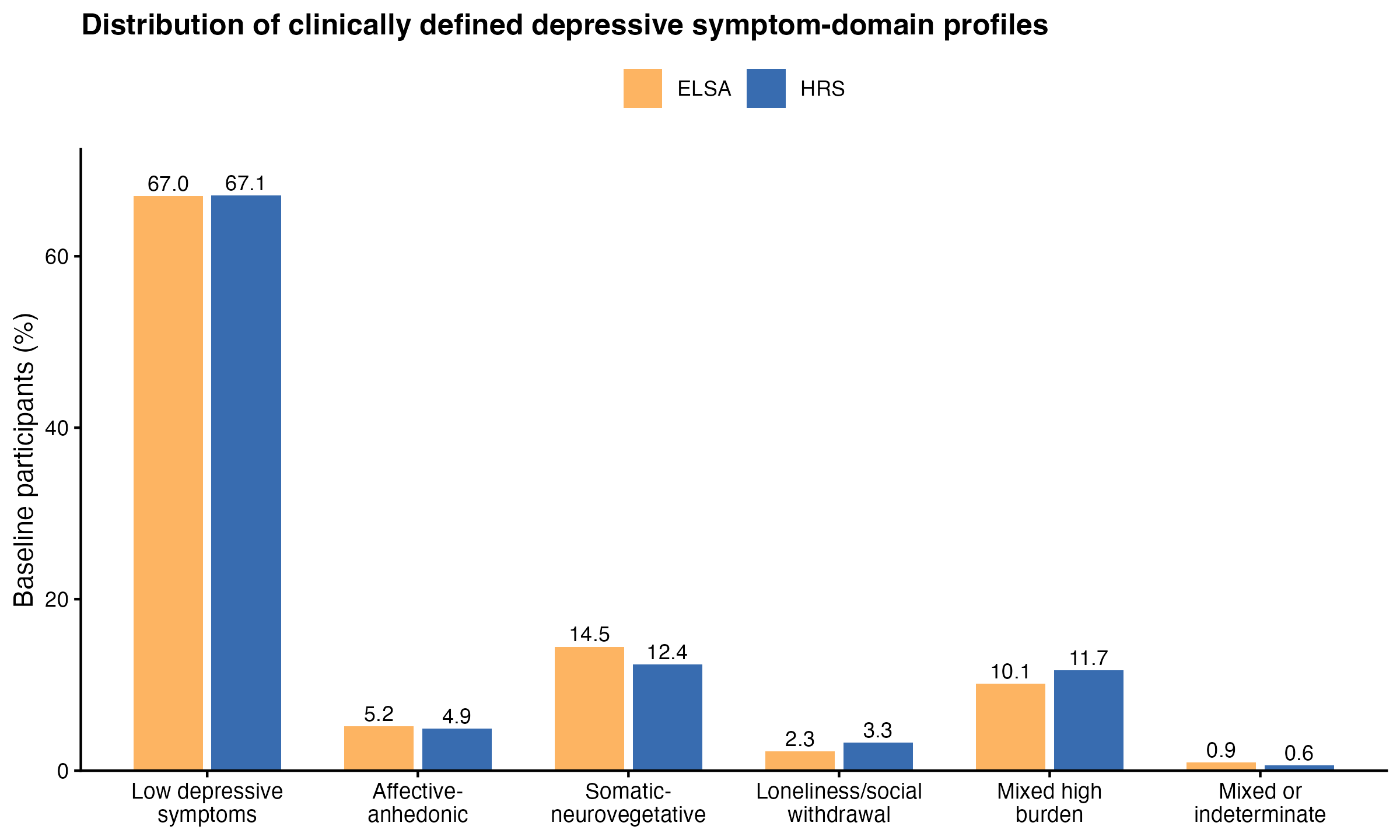

Supplement: Supplementary file 1 [file DataSheet1.zip › supplementary_figures/supplementary_figure1.png]

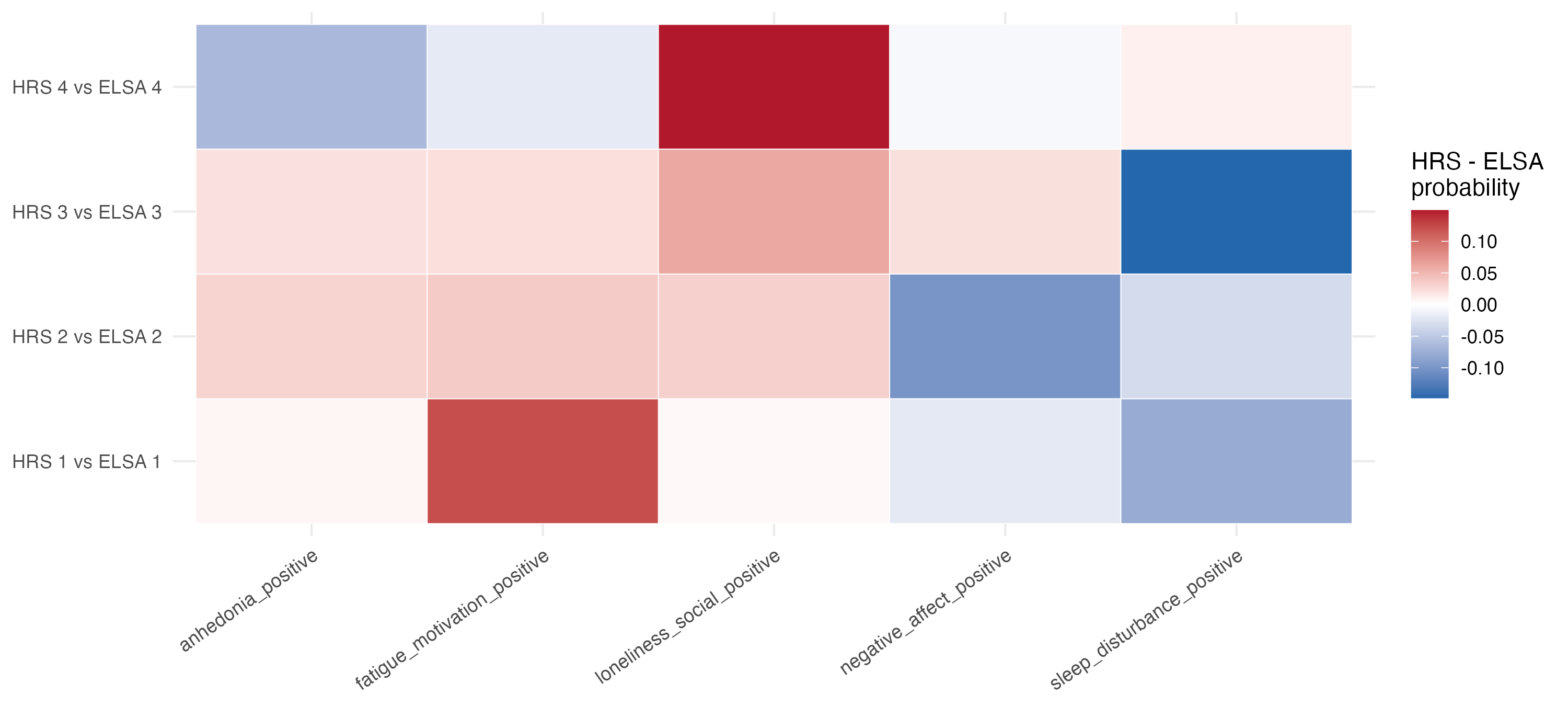

Supplement: Supplementary file 1 [file DataSheet1.zip › supplementary_figures/supplementary_figure2.png]

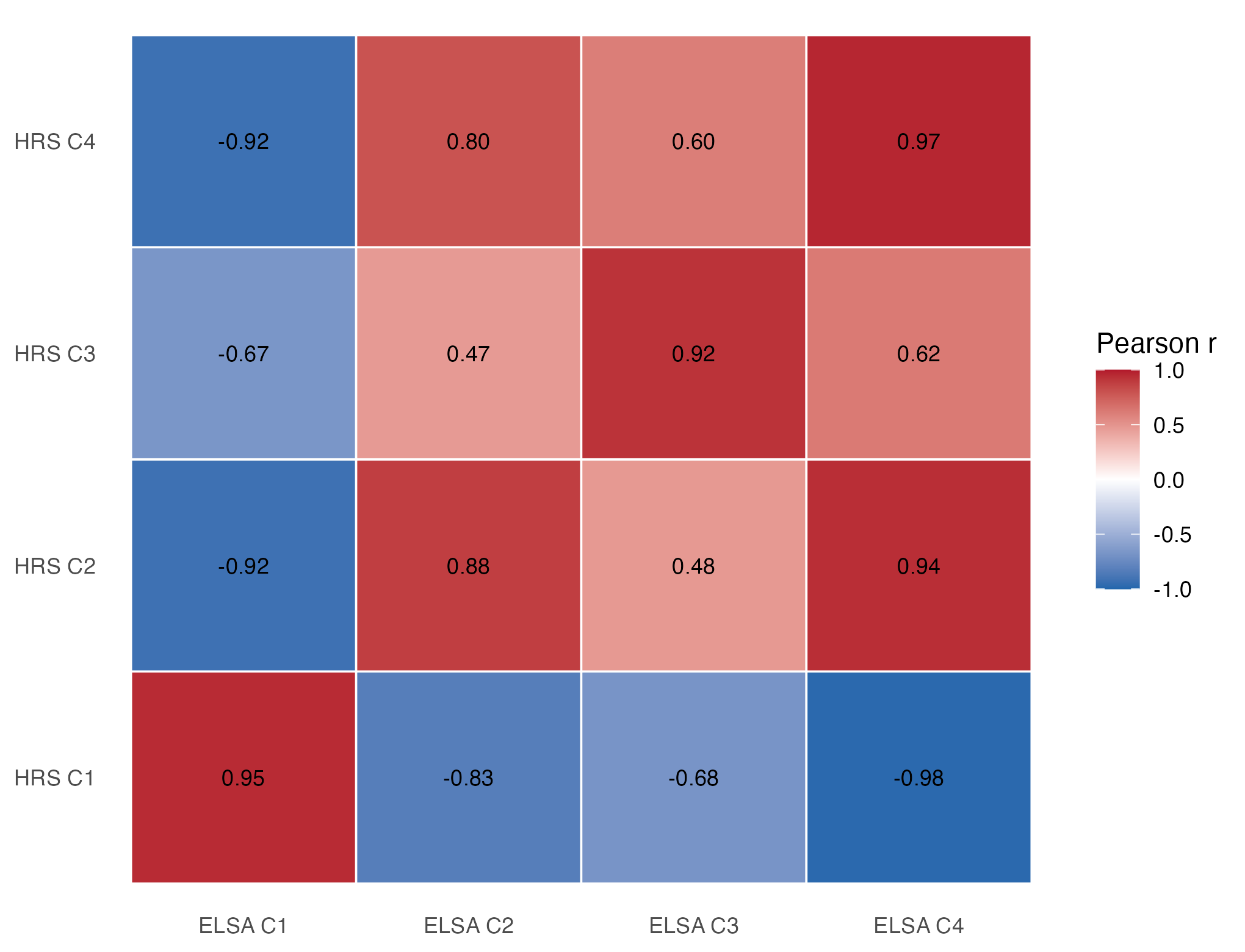

Supplement: Supplementary file 1 [file DataSheet1.zip › supplementary_figures/supplementary_figure3.png]

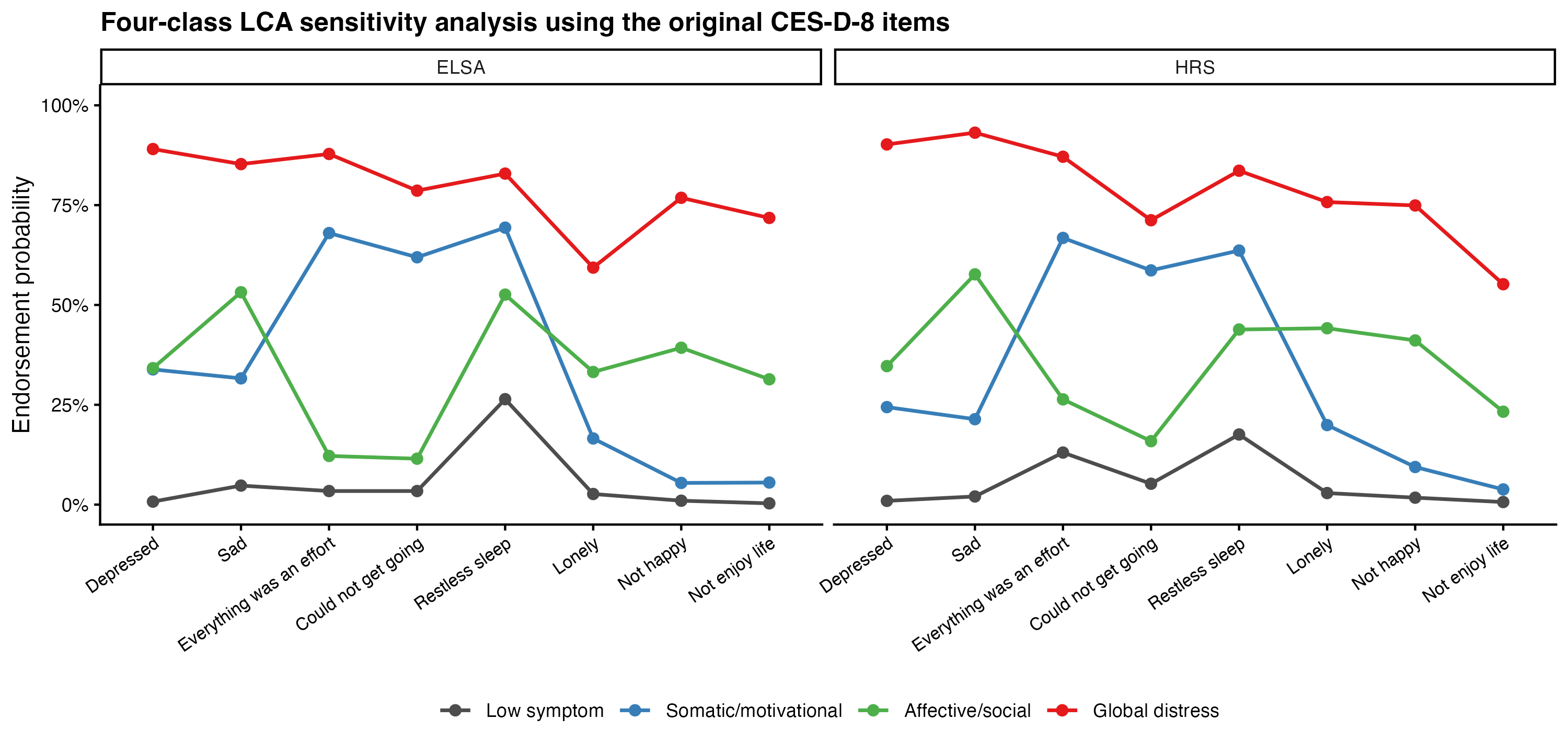

Supplement: Supplementary file 1 [file DataSheet1.zip › supplementary_figures/supplementary_figure5_cesd8_item_lca_k4.png]
